# Fragmented mitochondrial genomes are present in both major clades of the blood-sucking lice (suborder Anoplura): evidence from two *Hoplopleura* rodent lice (family Hoplopleuridae)

**DOI:** 10.1186/1471-2164-15-751

**Published:** 2014-09-02

**Authors:** Wen-Ge Dong, Simon Song, Xian-Guo Guo, Dao-Chao Jin, Qianqian Yang, Stephen C Barker, Renfu Shao

**Affiliations:** Institute of Pathogens and Vectors, Dali University, and Yunnan Provincial Key Laboratory for Zoonosis Control, Dali, China; Institute of Entomology, Guizhou University, and the Provincial Key Laboratory for Agricultural Pest Management in Mountainous Region, Guiyang, China; GeneCology Research Centre, Faculty of Science, Health, Education and Engineering, University of the Sunshine Coast, Maroochydore, Queensland Australia; College of Agronomy and Biotechnology, China Agricultural University, Beijing, China; Parasitology Section, School of Chemistry and Molecular Biosciences, University of Queensland, Queensland, Australia

**Keywords:** Mitochondrial genome, Genome fragmentation, Minichromosome, Chromosome evolution, Sucking lice

## Abstract

**Background:**

The suborder Anoplura contains 540 species of blood-sucking lice that parasitize over 840 species of eutherian mammals. Fragmented mitochondrial (mt) genomes have been found in the lice of humans, pigs, horses and rats from four families: Pediculidae, Pthiridae, Haematopinidae and Polyplacidae. These lice, eight species in total, are from the same major clade of the Anoplura. The mt genomes of these lice consist of 9–20 minichromosomes; each minichromosome is 1.5–4 kb in size and has 1–8 genes. To understand mt genome fragmentation in the other major clade of the Anoplura, we sequenced the mt genomes of two species of rodent lice in the genus *Hoplopleura* (family Hoplopleuridae).

**Results:**

We identified 28 mt genes on 10 minichromosomes in the mouse louse, *Ho. akanezumi*; each minichromosome is 1.7–2.7 kb long and has 1–6 genes. We identified 34 mt genes on 11 minichromosomes in the rat louse, *Ho. kitti*; each minichromosome is 1.8–2.8 kb long and has 1–5 genes. *Ho. akanezumi* also has a chimeric minichromosome with parts of two rRNA genes and a full-length tRNA gene for tyrosine. These two rodent lice share the same pattern for the distribution of all of the protein-coding and rRNA genes but differ in tRNA gene content and gene arrangement in four minichromosomes. Like the four genera of blood-sucking lice that have been investigated in previous studies, the *Hoplopleura* species have four minichromosomes that are only found in this genus.

**Conclusions:**

Our results indicate that fragmented mt genomes were present in the most recent common ancestor of the two major clades of the blood-sucking lice, which lived ~75 million years ago. Intra-genus variation in the pattern of mt genome fragmentation is common in the blood-sucking lice (suborder Anoplura) and genus-specific minichromosomes are potential synapomorphies. Future studies should expand into more species, genera and families of blood-sucking lice to explore further the phylogenetic utility of the novel features associated with fragmented mt genomes.

**Electronic supplementary material:**

The online version of this article (doi:10.1186/1471-2164-15-751) contains supplementary material, which is available to authorized users.

## Background

The suborder Anoplura contains 540 species of blood-sucking lice that parasitize over 840 species of eutherian mammals [1–3]. Blood-sucking lice are of medical and veterinary significance as parasites and vectors of disease agents [4–7]. According to Light et al. [8] and Smith et al. [9], the blood-sucking lice evolved from chewing lice ~92 million years ago (Mya) and diversified into two major clades ~75 Mya. One major clade includes the lice of bovids (family Linognathidae), rabbits, rodents and shrews (genera *Hoplopleura* and *Pterophthirus* of the family Hoplopleuridae; genera *Haemodipsus*, *Linognathoides*, *Neohaematopinus* and *Sathrax* of the family Polyplacidae), and sea lions and seals (family Echinophthiridae). The other major clade includes the lice of humans and gorillas (families Pediculidae and Pthiridae), monkeys (family Pedicinidae), pigs and horses (family Haematopinidae), and rodents (genus *Ancistroplax* of the family Hoplopleuridae; genera *Fahrenholzia*, *Polyplax* and *Lemurpediculus* of the family Polyplacidae). Like Kim [10], Light et al. [8] divided the blood-sucking lice into two major clades, although it differed from Kim [9] in the placement of the family Echinophthiridae, and in recognizing the families Hoplopleuridae and Polyplacidae as polyphyletic.

The mitochondrial (mt) genomes of eight species of blood-sucking lice have been sequenced entirely or near entirely in previous studies: 1) the human body louse, *Pediculus humanus* [11]); 2) the human head louse, *Pe. capitis*, and the human pubic louse, *Pthirus pubis* [12]; 3) the domestic pig louse, *Haematopinus suis*, and the wild pig louse, *Ha. apri* [13]; 4) the horse louse, *Ha. asini* [14]; and 5) the lice of the greater bandicoot rat and the Asian house rat, *Polyplax asiatica* and *Po. spinulosa* [15]. All of these lice have fragmented mt genomes that depart radically from the typical single-chromosome mt genomes seen in bilateral animals [16, 17]. The mt genes of these lice are on 9–20 minichromosomes; each minichromosome is 1.5–4 kb in size and has 1–8 genes. There is substantial variation in the extent of mt genome fragmentation and the distribution of mt genes over the minichromosomes among these lice [13]. Indeed, the fragmentation pattern varies even between species of the same genus [14, 15]. It is evident that recombination between minichromosomes played a role in generating the high degree of variation in the extent and the pattern of mt genome fragmentation among the blood-sucking lice [12, 14].

The three human lice (families Pediculidae and Pthiridae), the two pig lice and the horse louse (family Haematopinidae), and the two *Polyplax* rat lice (Polyplacidae), whose mt genomes have been sequenced, are from the same major clade of the Anoplura [8]. To understand mt genome fragmentation in the other major clade of the Anoplura, we sequenced the mt genomes of a mouse louse and a rat louse in the genus *Hoplopleura* (family Hoplopleuridae): *Ho. akanezumi* and *Ho. kitti*. Hoplopleuridae is the most species-rich family of the Anoplura with 162 described species, and *Hoplopleura* is the most species-rich genus in the family Hoplopleuridae with 141 described species found on rodents and pikas [2, 3, 8]. *Hoplopleura* species are in a major clade different from that of the human lice (families Pediculidae and Pthiridae), the pig lice and the horse louse (family Haematopinidae), and the *Polyplax* rat lice (Polyplacidae) [8]. *Ho. akanezumi* infests the Cheverier’s field mouse, *Apodemus chevrieri*, and five other *Apodemus* species of mice [3, 18] *. Ho. kitti* infests the Bower’s white-toothed rat, *Berylmys bowersi*, and two other species of rats, *Berylmys berdmorei* and *Leopoldamys edwardsi* [3, 18]. We found that both *Ho. akanezumi* and *Ho. kitti* have fragmented mt genomes. These two rodent lice, however, differ in the pattern of mt genome fragmentation. Like other genera of blood-sucking lice, the *Hoplopleura* species also have minichromosomes that are only found in this genus.

## Methods

### Collection of rodents and lice

The Chevrier’s field mouse lice, *Ho. akanezumi*, were collected at Cangshan Mountain, Dali city, Yunnan, China (sample No. 249). The Bower’s white-toothed rat lice, *Ho. kitti,* were collected in Jinping county, Yunnan, China (sample No. 344). The rodents were caught with trap-cages set outdoors (farmlands, scrublands and woodlands). Alive rodents trapped were placed individually in pre-marked cotton bags and transferred to laboratory for species identification and parasitological check. Blood-sucking lice on the body surface of each rodent host were collected and preserved in 95% ethanol at –20°C prior to DNA extraction. Samples of *Ho. akanezumi* and *Ho. kitti* and their rodent hosts were deposited in the Institute of Pathogens and Vectors, Dali University. The capture of rodents was approved by health authorities in Yunnan province, China. Animal protocols and procedures were approved by the animal ethics committees at Guizhou University and Dali University (2004C0049M, 30460125).

### DNA extraction, mitochondrial genome amplification and sequencing

**Figure 1:**
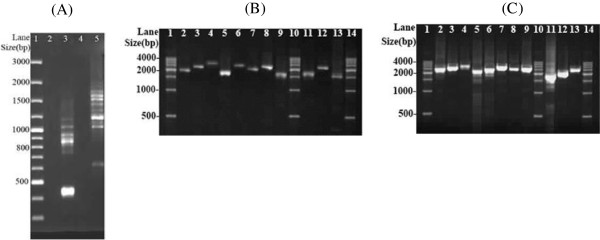
**PCR amplification of the mitochondrial (mt) minichromosomes of the** ***Hoplopleura*** **rodent lice. (A)** Lane 1: GeneRuler®100 bp DNA Ladder (Thermo Scientific). Lane 3: PCR amplicons generated with primer pair 249F-249R that spans the coding region of each mt minichromosome of *Hoplopleura akanezumi*. Lane 5: PCR amplicons generated with primer pair 344F-344R that spans the coding region of each mt minichromosome of *Hoplopleura kitti*. **(B)** PCR verification of the mt minichromosomes of *Ho. akanezumi*. Lane 1, 10 and 14: 500 bp DNA Ladder (TIANGEN). Lane 2-13: PCR amplicons from the 10 minichromosomes and a chimeric mt minichromosome of *Ho. akanezumi*: *atp8-atp6-* ***trnN***, *trnE-cob-* ***trnS***_***1***_ *-trnS*_*2*_, *trnI-* ***cox1***, *trnD-trnY*_*1*_ *-* ***cox2***, *trnR-nad4L-trnP-* ***cox3*** *-trnA-trnT*, ***nad2***, *trnK-* ***nad4***, *trnC-* ***nad6*** *-trnW-trnL*_*2*_ *(taa)*, ***rrnS***, *trnY*_*2*_ *-* ***rrnL*** *-trnV*, chimeric *trnY*_*2*_ *-* ***prrnL*** *-* ***prrnS***. **(C)** PCR verification of the mt minichromosomes of *Ho. kitti*. Lane 1, 10 and 13: 500 bp DNA Ladder (TIANGEN). Lane 2-13: PCR amplicons from the 11 minichromosomes of *Ho. kitti*: *atp8-atp6-* ***trnN***, ***trnE*** *-cob-trnS*_*1*_ *-trnS*_*2*_, ***trnI*** *- cox1*, *trnD-trnY-* ***cox2*** *-trnT*, *trnR-nad4L-trnP-* ***cox3*** *-trnA*, *trnQ-* ***nad1*** *-trnG-nad3*, ***nad2***, ***trnK*** *-nad4*, *trnC-* ***nad6*** *-trnW-trnL*_*2*_ *(taa)*, ***rrnS***, *trnM-trnL*_*1*_ *(tag)-* ***rrnL*** *-trnV*. Genes from which PCR primers were designed are in bold.

Total DNA was extracted from individual louse specimens with DNeasy Tissue kit (QIAGEN). A 452-bp fragment of mt *rrnS* gene and a 360-bp fragment of mt *rrnL* gene were initially amplified by polymerase chain reaction (PCR) with primer pairs 12SA–12SB and 16SF–Lx16SR (Additional file 1) for *Ho. akanezumi*. These two pairs of primers target conserved sequence motifs that are highly conserved among arthropods. The *rrnS* and *rrnL* fragments were sequenced directly using Sanger method at the Tiangen Biotech, Beijing (TBB). Two pairs of specific primers for *Ho. akanezumi*, 12S249F–12S249R and 16S249F–16S249R, were designed from sequences of the *rrnS* and *rrnL* fragments. The two specific primers in each pair go outbound and are 1 bp and 89 bp respectively from each other. PCRs with these specific primers amplified two near full-length mt minichromosomes of *Ho. akanezumi* that contain *rrnS* and *rrnL* respectively; these amplicons (1.7 kb and 2.1 kb in size) were sequenced using Sanger method at the TBB. Another pair of primers specific to *Ho. akanezumi*, 249F–249R, was designed from conserved non-coding sequences that flank the coding regions of the two minichromosomes above. The PCR with 249F–249R primers produced a mixture of amplicons ranging from 0.4 to 2 kb in size, expected from the coding regions of the whole set of mt minichromosomes of *Ho. akanezumi* (Figure 1A). These amplicons were sequenced with Illumina Hiseq 2000 platform at the BGI Hong Kong. The PCR strategy used in this study was developed from the observations we made in previous studies on the human lice, the pig lice, the *Polyplax* rat lice and the horse louse that each mt minichromosome has a distinct coding region but a well-conserved non-coding region [11–15].

For *Ho. kitti*, a 532-bp fragment of mt *cox1* gene, a 452-bp fragment of mt *rrnS* gene and a 360-bp fragment of mt *rrnL* gene were amplified initially by PCR with primer pairs mtd6–mtd11, 12SA–12SB and 16SF–Lx16SR (Additional file 1). These three pairs of primers target conserved sequence motifs in *cox1*, *rrnS* and *rrnL*; the PCR amplicons were sequenced using Sanger method at the TBB. Three pairs of specific primers, cox344F–cox344R, 12S344F–12S344R and 16S344F–16S344R were designed from sequences of the *cox1*, *rrnS* and *rrnL* fragments respectively. The specific primers in each pair go outbound and are 2 bp, 32 bp and 49 bp respectively from each other. PCRs with these three pairs of specific primers amplified three near full-length mt minichromosomes of *Ho. kitti* that contain *cox1*, *rrnS* and *rrnL* respectively; these amplicons (2.7 kb, 2.0 kb and 2.4 kb in size) were sequenced using Sanger method at the TBB. Another pair of primers specific to *Ho. kitti*, 344F–344R, was designed from conserved non-coding sequences that flank the coding regions of the three minichromosomes above. The PCR with primer pair 344F–344R produced a mixture of amplicons ranging from 0.6 to 2 kb in size, expected from the coding regions of all mt minichromosomes of *Ho. kitti* (Figure 1A). These amplicons were sequenced with Illumina Hiseq 2000 platform at the BGI-HK.

*Taq* DNA Polymerase (Tiangen Biotech) was used in the initial short PCRs with the following cycling conditions: 94°C for 1 min; 40 cycles of 98°C for 10 sec, 45°C for 30 sec, 72°C for 1 min; and a final extension of 72°C for 2 min. LA *Taq* (TakaRa) was used in the long PCRs with the cycling conditions: 94°C for 1 min; 35 cycles of 98°C for 10 sec, 60–65°C (depending on primers) for 30–40 sec, 68°C for 3 min; and a final extension of 72°C for 6 min. Positive and negative controls were run with each PCR experiment. PCR amplicons were checked by agarose gel (1%) eletrophoresis; the sizes of PCR amplicons were estimated by comparing with molecular markers. PCR products were purified with Wizard SV Gel/PCR clean-up system (Promega).

### Assembly of Illumina sequence-reads, gene identification and verification of individual mitochondrial minichromosomes

Purified PCR amplicons generated above with primers 249F–249R and 344F–344R from the coding regions of the mt minichromosomes of *Ho. akanezumi* and *Ho. kitti* were sequenced with Illumina Hiseq 2000 platform at the BGI-HK. Illumina sequence-reads were assembled into contigs with Geneious 6.1.7 [19]. The assembled parameters were minimum overlap identity 98% and minimum overlap 50 bp. tRNA genes were identified using tRNAscan-SE [20] and ARWEN [21]. Protein-coding genes and rRNA genes were identified with Basic Local Alignment Search Tool (BLAST) searches of GenBank [22, 23]. Identical sequences shared between genes were identified with Wordmatch [24]. Sequence alignments were with Clustal X [25]. The size and circular organization of each mt minichromosome of *Ho. akanezumi* and *Ho. kitti* identified by sequence-read assembly were verified by PCR (Figure 1B, C) using outbound primers designed from the coding region of each minichromosome (Additional file 2). The forward primer and reverse primer in each pair were next to each other with a small gap or no gap in between. PCRs with these primers amplified each minichromosome in full or near full length if it had a circular organization. PCR set-up, cycling conditions, agarose gel electrophoresis and size measurement were the same as described above. Positive and negative controls were run for all PCR tests. The nucleotide sequences of the mt genomes of *Ho. akanezumi* and *Ho. kitti* have been deposited in GenBank under accession numbers KJ648922-KJ648943.

## Results

### Mitochondrial genome of *Hoplopleura akanezumi*, the louse of the Chevrier’s field mouse

**Figure 2:**
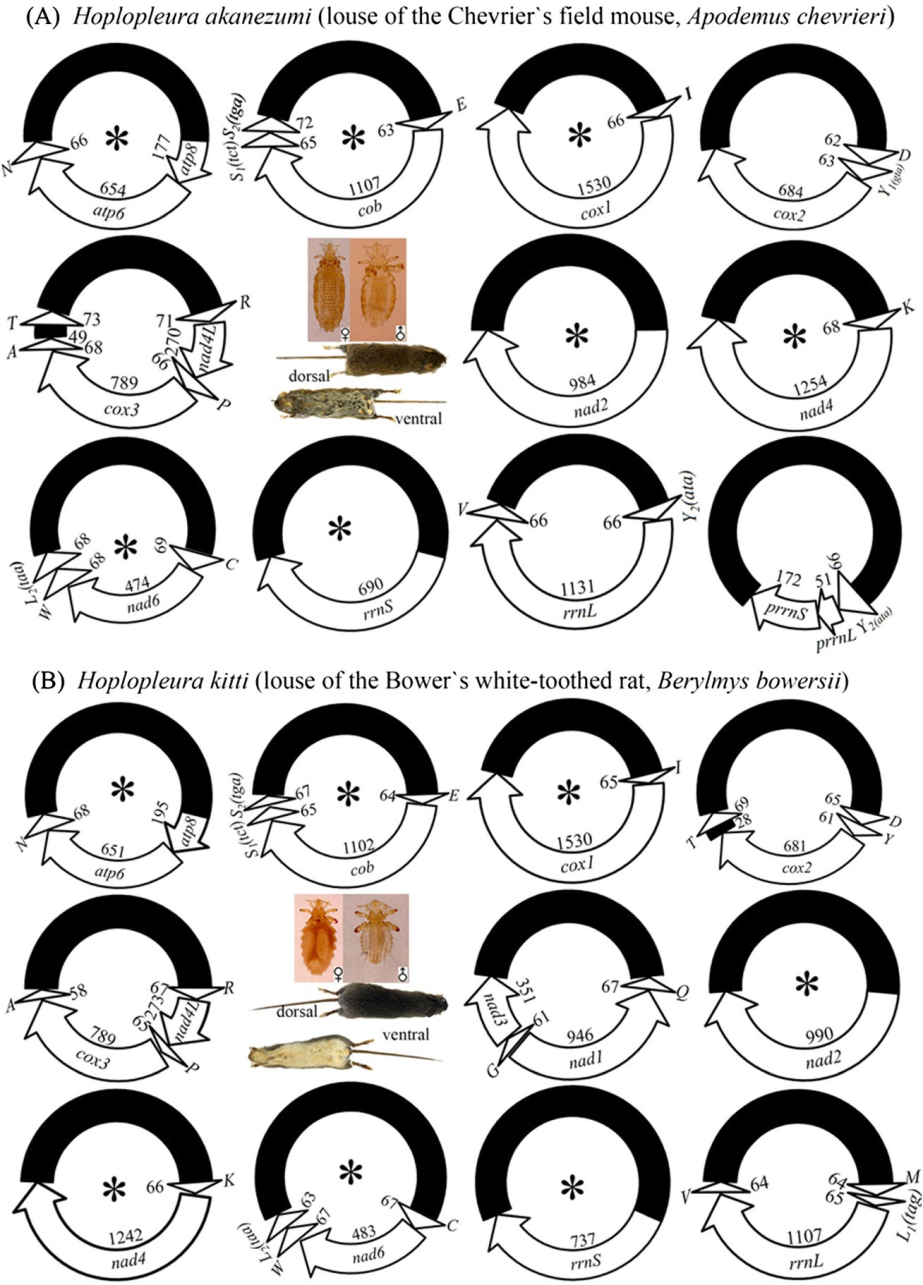
**The mitochondrial (mt) genomes of** ***Hoplopleura akanezumi*** **(A) and** ***Hoplopleura kitti*** **(B).** Each minichromosome has a coding region (with gene name, transcription orientation and length indicated) and a non-coding region (in black). Minichromosomes are in alphabetical order by the names of their protein coding and rRNA genes. Abbreviations of gene names are: *atp6* and *atp8* (for ATP synthase subunits 6 and 8), *cox1-3* (for cytochrome coxidase subunits 1-3), *cob* (for cytochrome b), *nad1-4* and *nad4L* (for NADH dehydrogenase subunits 1-6 and 4 L), *rrnS* and *rrnL* (for small and large subunits of ribosomal RNA). tRNA genes are shown with the single-letter abbreviations of their corresponding amino acids. Minichromosomes that have identical gene content and gene arrangement between the two *Hoplopleura* species are indicated with asterisk symbols “*”.

We obtained 609,880 sequence-reads from the mt genome of *Ho. akanezumi* with Illumina Hiseq platform (Table 1). These sequence-reads are 90 bp long each. We assembled the sequence-reads into contigs and identified 28 of the 37 mt genes that are typical of bilateral animals in *Ho. akanezumi*; these 28 genes are on 10 minichromosomes. Each minichromosome is 1.7–2.6 kb in size, consists of a coding region and a non-coding region, and has a circular organization (Figure 1B). The coding region of each minichromosome contains one to six genes, and varies in size from 678 bp for *trnC-nad6-trnW-trnL*_*2*_ minichromosome to 1,596 bp for *trnI-cox1* minichromosome (Table 1; Figure 2A) (Note: minichromosomes are named after their genes hereafter). Eight of the 10 minichromosomes of *Ho. akanezumi* have one protein-coding or rRNA gene each; the other two minichromosomes have two protein-coding genes each. The 16 tRNA genes we identified are on eight of the 10 minichromosomes; each minichromosome has one to four tRNA genes except *nad2* minichromosome and *rrnS* minichromosome, which have no tRNA genes (Figure 2A; Additional file 3). Each of the 28 mt genes identified in *Ho. akanezumi* is present on only one minichromosome except *trnY*_*2*_, which is also present in the chimeric minichromosome (see below). All of the 28 mt genes of *Ho. akanezumi* that we found have the same orientation of transcription relative to the non-coding region (Figure 2A).

**Table 1:** **Mitochondrial minichromosomes of** ***Hoplopleura akanezumi*** **and** ***Hoplopleura kitti*** **identified by Illumina sequencing**

Minichromosome	Size of coding region (bp)	Number of Illumina sequence-reads
*atp8-atp6-N (atp8-atp6-N)*	898 (924)	31926 (99377)
*E-cob-S*_*1*_ *-S*_*2*_ *(E-cob-S*_*1*_ *-S*_*2*_ *)*	1309 (1304)	2539 (52216)
*I-cox1 (I-cox1)*	1596 (1644)	6545 (35537)
*D-Y*_*1*_ *-cox2 (D-Y-cox2-T)*	810 (904)	55030 (42531)
*R-nad4L-P-cox3-A-T (R-nad4L-P-cox3-A)*	1390 (1250)	1706 (59511)
*(Q-nad1-G-nad3)*	(1447)	(51409)
*nad2 (nad2)*	984 (990)	22613 (50604)
*K-nad4 (K-nad4)*	1322 (1317)	2059 (41629)
*C-nad6-W-L*_*2*_ *(C-nad6-W-L*_*2*_ *)*	678 (685)	33296 (60815)
*rrnS (rrnS)*	690 (695)	166399 (88891)
*Y*_*2*_ *-rrnL-V (M-L*_*1*_ *-rrnL-V)*	1263 (1299)	50213 (60799)
chimeric *Y*_*2*_ *-prrnS-prrnL*	433	237554
Total	11373 (12459)	609880 (643319)

Note: Gene arrangements and numbers outside brackets are for *Hoplopleura akanezumi* and those in brackets are for *Hoplopleura kitti.*

We found a chimeric minichromosome in *Ho. akanezumi* (Figure 2A). This chimeric minichromosome consists of a coding region and a non-coding region, and has a circular organization (Figure 1B). The coding region of the chimeric minichromosome is 289 bp and contains parts of the two rRNA genes, *prrnL* and *prrnS* (note: *p* for partial), which are only 5% (i.e. 51 bp) and 24% (i.e. 172 bp) of the full-length *rrnL* and *rrnS*, respectively. A tRNA gene, *trnY*_*2*_, is upstream *prrnL* in the chimeric minichromosome and has the same sequence and length as its counterpart in the *trnY*_*2*_*-rrnL-trnV* minichromosome (Figure 2A, Table 1).

**Figure 3:**
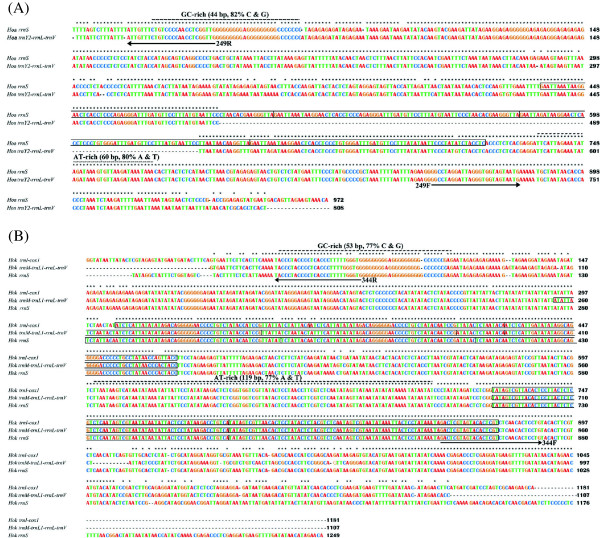
**Alignment of nucleotide sequences in the non-coding regions of the mitochondrial minichromosomes of** ***Hoplopleura akanezumi*** **(A) and** ***Hoplopleura kitti*** **(B).** 249F and 249R are the primers used to amplify the coding regions of all mitochondrial minichromosomes of *Ho. akanezumi*. 344F and 344R are the primers used to amplify the coding regions of all mitochondrial minichromosomes of *Ho. kitti*.

We sequenced the full-length non-coding regions of the *rrnS* minichromosome and *trnY*_*2*_*-rrnL-trnV* minichromosome of *Ho. akanezumi*, 972 bp and 808 bp long, respectively (Figure 3A). The non-coding regions of these two minichromosomes have 78.7% identity to each other. As in other sucking lice [11–15], an AT-rich motif (60 bp, 80% A and T) is present in the non-coding regions of *Ho. akanezumi* upstream the 5’-end of the coding region, whereas a GC-rich motif (44 bp, 82% G and C) is present downstream the 3’-end of the coding region (Figure 3A). A 72-bp motif repeated four times in tandem in the NCR of *rrnS* minichromosome of *Ho. akanezumi*; the four repeat units have 94% identity to each other. No tandem repetitive sequences were found in the NCR of *trnY*_*2*_*-rrnL-trnV* minichromosome. In addition to the full-length NCR sequences of the two minichromosomes, we also sequenced parts of the NCRs of the other eight minichromosomes and the chimeric minichromosome upstream and downstream of the coding regions, 74–121 bp and 37–128 bp respectively (Additional file 4). Two highly conserved sequence-motifs, 74 bp and 37 bp long respectively, are present in the sections of the NCRs upstream and downstream the coding regions of all of the minichromosomes and the chimeric minichromosome of *Ho. akanezumi* (Additional file 4).

### Mitochondrial genome of *Hoplopleura kitti*, the louse of the Bower’s white-toothed rat

We obtained 643,319 sequence-reads from the mt genome of *Ho. kitti* by Illumina sequencing (Table 1). As above for *Ho. akanezumi*, these sequence-reads are 90 bp each in length. We assembled these sequence-reads into contigs and identified 34 mt genes typical of bilateral animals in *Ho. kitti*. These genes are on 11 minichromosomes; each minichromosome is 1.8–2.8 kb in size, has a circular organization and consists of a coding region and a non-coding region (Figure 2B; Figure 1C). As in *Ho. akanezumi*, eight of the 11 minichromosomes of *Ho. kitti* have one protein or rRNA-coding gene each; the other three minichromosomes have two protein-coding genes each. The 19 tRNA genes are on nine of the 11 minichromosomes; each minichromosome has one to three tRNA genes except *rrnS* minichromosome and *nad2* minichromosome, which have no tRNA genes (Figure 2B; Additional file 3). Each minichromosome has a coding region and a non-coding region. The coding region of each minichromosome contains one to five genes, and varies in size from 685 bp for *trnC-nad6-trnW-trnL*_*2*_ minichromosome to 1,644 bp for *trnI-cox1* minichromosome. With the exception of *nad1* and *trnQ*, all of the mt genes of *Ho. kitti* have the same orientation of transcription relative to the NCRs (Figure 2B).

We sequenced the full-length NCRs of three mt minichromosomes of *Ho. kitti*: *rrnS, trnM-trnL*_*1*_*-rrnL-trnV* and *trnI-cox1*, 1,249 bp, 1,107 bp and 1,181 bp respectively. The NCRs of these three minichromosomes have 87–93% identity to each other. Tandem repetitive sequences were found in the NCRs of all three minichromosomes. A 60-bp motif repeated three times in tandem; these three repeat units have 87–90% identity to each other. A 72-bp motif repeated twice in tandem; the two repeat units have 93% identity to each other.

As above in *Ho. akanezumi* and other sucking lice, an AT-rich motif (119 bp, 76% A and T) is present in the NCRs upstream the 5’-end of the coding region, and a GC-rich motif (50 bp, 79% G and C) is present downstream the 3’-end of the coding region in *Ho. kitti* (Figure 3B). Additional to the full-length NCR sequences, we also sequenced parts of the NCRs upstream and downstream of the coding regions of the other eight minichromosomes of *Ho. kitti*, 140–326 bp and 48–449 bp respectively. Two highly conserved sequence-motifs, 140 bp and 48 bp long respectively, are present in the sections of the non-coding regions upstream and downstream of the coding regions of all of the 11 minichromosomes in *Ho. kitti* (Additional file 5).

## Discussion

### Intra-genus variation in the pattern of mt genome fragmentation in blood-sucking lice

The two species of *Hoplopleura* rodent lice have the same pattern for the distribution of all of the protein-coding and rRNA genes and most tRNA genes on their minichromosomes (Figure 2). Each minichromosome has a single protein-coding or rRNA gene except for three minichromosomes that have two protein-coding genes each: *atp8*–*atp6*, *nad4L*–*cox3*, and *nad1*–*nad3* (Figure 2). Seven minichromosomes of *Ho. akanezumi* and *Ho. kitti* have identical gene content and gene arrangement; the other four minichromosomes, however, differ between the two species, providing further evidence for intra-genus variation in the pattern of mt genome fragmentation in the blood-sucking lice. Two recent studies also revealed variation in the pattern of mt genome fragmentation within two other genera of the blood-sucking lice. Dong et al. [15] showed that two *Polyplax* species of rat lice differ in gene content and gene arrangement for nine of the 11 mt minichromosomes; the variation, however, was limited to tRNA genes only. Song et al. [14] showed that the horse louse, *Haematopinus asini*, differs from the pig lice, *Ha. suis* and *Ha. apri*, in gene content and gene arrangement for three of the nine minichromosomes; the variation is not limited to tRNA genes but in protein-coding and rRNA genes as well. Taken together, we conclude that intra-genus variation in the pattern of mt genome fragmentation is common in both major clades of the blood-sucking lice; such variation may involve tRNA genes, protein-coding genes and rRNA genes.

### What is the ancestral pattern of mt genome fragmentation of the blood-sucking lice?

According to Light et al. [8] and Smith et al. [9], the blood-sucking lice (suborder Anoplura) evolved from chewing lice ~92 Mya and diversified into two major clades ~75 Mya. The *Hoplopleura* species, which we investigated in the current study, are in the major clade with the lice of bovids (family Linognathidae), rabbits and shrews (genus *Pterophthirus* of the family Hoplopleuridae; genera *Haemodipsus*, *Linognathoides*, *Neohaematopinus* and *Sathrax* of the family Polyplacidae), and sea lions and seals (family Echinophthiridae). The other major clade includes the lice of humans and gorillas (families Pediculidae and Pthiridae), monkeys (family Pedicinidae), pigs and horses (family Haematopinidae), and rodents (genus *Ancistroplax* of the family Hoplopleuridae; genera *Fahrenholzia*, *Polyplax* and *Lemurpediculus* of the family Polyplacidae). Light et al. [8] and Smith et al. [9] used molecular data (*18S*, *EF-1α*, and *cox1* gene sequences) of species from eight of the 15 families of the Anoplura and is consistent with an early study by Kim [10], which used morphological data, in dividing the blood-sucking lice into two major clades.

The presence of fragmented mt genomes in both major clades of the blood-sucking lice indicates that the most recent common ancestor (MRCA) of these lice already had a fragmented mt genome. Although the data available now are not sufficient yet for us to establish the exact ancestral fragmentation pattern of that MRCA, we can infer that the gene-arrangement characters that are present in the *Hoplopleura* species and the species from the other major clade to be ancestral to the blood-sucking lice. These characters include *atp8-atp6-N*, *E-cob*, *cob-S*_*1*_, *I-cox1*, *D-Y-cox2*, *R-nad4L-P-cox3*, *cox3-A*, *K-nad4*, and *M-L*_*1*_*-rrnL-V*; each of these characters is shared by at least one species from each of the two major clades of the blood-sucking lice (Table 2).

**Table 2:** **Mitochondrial gene-arrangement characters inferred to be ancestral for blood- sucking lice (suborder Anoplura)**

Species of insects	Order/suborder	***atp8-atp6-N***	***E-cob***	***cob-S***_***1***_	***I-cox1***	***D-Y-cox2***	***R-nad4L-P-cox3***	***cox3-A***	***K-nad4***	***M-L***_***1***_ ***-rrnL-V***
*Hoplopleura akanezumi* (rat louse)	Phthiraptera/Anoplura	+	+	+	+	+	+	+	+	–
*Hoplopleura kitti* (rat louse)	Phthiraptera/Anoplura	+	+	+	+	+	+	+	+	+
*Pediculus capitis* (human head louse)	Phthiraptera/Anoplura	–	–	–	–	–	–	+	+	–
*Pediculus humanus* (human body louse)	Phthiraptera/Anoplura	–	–	–	–	–	–	+	+	–
*Pthirus pubis* (human pubic louse)	Phthiraptera/Anoplura	–	–	+	–	–	–	+	–	–
*Polyplax asiatica* (rat louse)	Phthiraptera/Anoplura	–	+	–	–	+	–	+	+	+
*Polyplax spinulosa* (rat louse)	Phthiraptera/Anoplura	–	+	–	–	+	+	–	+	–
*Haematopinus suis* (domestic pig louse)	Phthiraptera/Anoplura	+	+	–	+	+	–	+	+	–
*Haematopinus apri* (wild pig louse)	Phthiraptera/Anoplura	+	+	–	+	+	–	+	+	–
*Haematopinus asini* (horse louse)	Phthiraptera/Anoplura	+	+	–	+	+	–	+	+	–
*Bothriometopus macrocnemis* (screamer louse)	Phthiraptera/Ischnocera	–	–	–	–	–	–	–	+	–
*Campanulotes bidentatus* (pigeon louse)	Phthiraptera/Ischnocera	–	–	–	–	–	–	–	–	–
*Ibidoecus bisignatus* (ibis head louse)	Phthiraptera/Ischnocera	–	–	–	–	–	–	–	–	–
*Heterodoxus macropus* (wallaby louse)	Phthiraptera/Amblycera	–	–	–	–	–	–	–	–	–
Lepidopsocid sp. (barklouse)	Psocoptera/Trogiomorpha	–	–	–	–	–	–	–	–	–
*Haematomyzus elephantis* (elephant louse)	Phthiraptera/ Rhynchophthirina	–	–	–	+	–	–	–	+	–
Hypothetical ancestor of insects		–	–	–	–	–	–	–	–	–

Note: “+” is for “presence”; “-” is for “absence”.

### Recombination between mt genes and between mt minichromosomes in *Hoplopleura*lice

Evidence for recombination between mt genes and between mt minichromosomes has been found in human lice, pig lice, a horse louse, and *Polyplax* rat lice in previous studies. In the human lice, *Pe. humanus*, *Pe. capitis* and *Pt. pubis*, eight pairs of mt genes share stretches of identical sequences, 16–127 bp long, which are much longer than expected by chance, providing unequivocal evidence for recombination between mt genes [11, 12, 26]. Longer-than-expected identical sequences shared between mt genes were also found in the pig lice, *Ha. suis* and *Ha. apri* (three pairs of genes [13]), the horse louse, *Ha. asini* (nine pairs of genes [14]), and the rat lice, *Po. asiatica* (one pair of genes) and *Po. spinulosa* (three pairs of genes [15]). It is noteworthy that *trnL*_*1*_ and *trnL*_*2*_ share identical sequences that are much longer than expected by chance in all of these five blood-sucking lice. In the three species of human lice, *trnL*_*1*_ and *trnL*_*2*_ have identical sequences except for the nucleotides at the third anti-codon positions [12]. In the two species of pig lice and the two species of *Polyplax* rat lice, these two tRNA genes have near identical sequences at the D-arm and the AC-arm except for the nucleotides at the third anti-codon positions, but differ at the T-arm and the AA-arm [13, 15]. In the horse louse, *trnL*_*1*_ and *trnL*_*2*_ share a 15-bp identical sequence at the D-arm [14]. In addition, seven chimeric mt minichromosomes were found in the human body louse, *Pe. humanus*, providing evidence for recombination between mt minichromosomes [26, 27].

We did not find identical sequences shared by any mt genes, even between *trnL*_*1*_ and *trnL*_*2*_, longer than expected by chance in *Ho. akanezumi* and *Ho. kitti* (Table 3). *trnL*_*1*_ and *trnL*_*2*_, both of which were found in *Ho. kitti*, share only 7 bp identical sequence, which is expected by chance. Thus, there is no evidence for recombination between mt genes in these two *Hoplopleura* species. We found, however, a chimeric mt minichromosome in *Ho. akanezumi*, which has part of *rrnS* gene (*prrnS*, 172 bp; “*p*” for “partial” hereafter) and part of *rrnL* gene (*prrnL*, 51 bp) (Figure 2). The chimeric mt minichromosome of *Ho. akanezumi* has similar structure to the Type 1 and Type 2 chimeric minichromosomes of the human body louse [26]. Type 1 chimeric minichromosome of the human body louse has *pcox2* and *pcox3*, whereas Type 2 has *patp6* and *pnad1* [26]. The partial genes in each of these chimeric minichromosomes of the human body louse were joined at a short homologous sequence, called “microhomology”, a hallmark of the gene junction formed by non-homologous recombination [26, 28, 29]. Intriguingly, we did not find such microhomology in the junction between *prrnS* and *prrnL* in the chimeric minichromosome of *Ho. akanezumi*. It is not clear yet to us whether, or not, the lack of recombination between mt genes in the two *Hoplopleura* lice and the lack of microhomology in the chimeric minichromosome of *Ho. akanezumi* are a general feature for the major clade of blood-sucking lice that they represent. More species from this major clade need to be investigated to understand the recombination between mt genes and between mt minichromosomes.

**Table 3:** **The longest stretches of identical sequence shared by mitochondrial genes in four rat lice, two pig lice, three human lice that have fragmented mitochondrial genomes, and six other species of bilateral animals that have the typical mitochondrial genomes**

Pairs of gene		The longest stretches of identical sequence														
		Rodent lice				Pig lice		Human lice			Animals with typical mt genome organization					
		***Hoa***	***Hok***	***Pa***	***Ps***	***Has***	***Haa***	***Pc***	***Pp***	***Ph***	***Bm***	***Cb***	***Hm***	***Dy***	***Ce***	***Hos***
*trnL*_*1*_	*trnL*_*2*_	NA	**7**	**28**	**25, 11**	**16, 10**	**16, 10**	**33, 32**	**35, 32**	**33, 32**	7	6	7	10	6	6
*cob*	*nad5*	NA	NA	12	**36**	13	13	12	13	12	12	16	14	13	13	12
*trnA*	*trnC*	7	6	6	**32**	6	6	6	6	6	NA	7	17	7	8	6
*nad4*	*nad5*	NA	NA	12	**18**	12	12	**127, 30**	NA	**127, 30**	13	15	15	16	14	11
*nad5*	*rrnL*	NA	NA	13	13	11	10	**99**	10	**99**	12	14	13	15	16	10
*trnG*	*trnR*	NA	6	5	5	5	5	**28, 14**	**32, 26**	**28, 14**	5	6	7	6	8	6
*cox1*	*nad4L*	10	10	9	11	11	11	10	**29**	10	13	11	14	13	12	10
*nad2*	*rrnL*	11	11	10	11	10	11	**26**	10	**26**	13	11	14	13	12	10
*trnP*	*trnT*	7	8	6	7	**26**	**26**	7	NA	7	6	8	8	9	10	7
*atp8*	*trnG*	NA	7	6	6	6	6	**26**	9	**26**	10	11	11	12	NA	6
*atp8*	*nad2*	9	9	12	10	**25**	**25**	10	8	10	10	14	12	14	NA	11
*trnI*	*trnT*	6	6	10	8	6	6	6	**16**	6	6	5	7	7	9	6

Note: Abbreviations of species names are: *Hoa, Hoplopleura akanezumi* (louse of the Chevrier’s field mouse); *Hok, Hoplopleura kitti* (louse of the Bower’s white-toothed rat); *Pa*, *Polyplax asiatica* (loues of the greater bandicoot rat); *Ps*, *Polyplax spinulosa* (louse of the Asian house rat); *Has*, *Haematopinus suis* (domestic pig louse); *Haa*, *Haematopinus apri* (wild pig louse); *Pc*, *Pediculus capititis* (human head louse); *Pp*, *Pthirus pubis* (human pubic louse); *Ph*, *Pediculus humanus* (human body louse); *Bm*, *Bothriometopus macrocnemis* (screamer louse); *Cb, Campanulotes bidentatus* (pigeon louse); *Hm*, *Heterodoxus macropus* (wallaby louse); *Dy*, *Drosophila yakuba* (fruitfly); *Ce*, *Caenorhabditis elegans* (roundworm); *Hos*, *Homo sapiens* (human); NA, not applicable. Stretches of shared identical sequences longer than expected by chance are indicated in bold.

### Are features of fragmented mt genomes useful for understanding phylogeny?

The suborder Anoplura contains 540 species of blood-sucking lice in 50 genera and 15 families [1–3]. The monophyly of the suborder Anoplura was inferred from morphology and has been generally accepted [30]. The monophyly of most of the genera and families, however, has not been tested. Furthermore, phylogenetic relationships among most of the genera and families are not resolved or are controversial. Indeed, only four studies have addressed the high-level phylogenetic relationships, i.e. among genera and among families of the blood-sucking lice: Kim and Ludwig [1] and Kim [10] used morphological characters, whereas Light et al. [8] and Smith et al. [9] used molecular data (*18S*, *EF-1α*, and *cox1* gene sequences). As discussed above, the findings from the present study and two recent studies [14, 15] showed that intra-genus variation in the pattern of mt genome fragmentation is common among blood-sucking lice. Such variation may provide a novel source of information, additional to morphology and gene sequences, for inferring genus- and family-level phylogenies of the blood-sucking lice, and for testing the phylogenies inferred from morphology and gene sequences. Along this line, we found that each of the five genera of the blood-sucking lice that have been investigated has one to nine minichromosomes that are only present in that genus (Table 4), which could potentially be synapomorphies for these genera. Future studies should expand into other genera and families of the blood-sucking lice to fully evaluate the phylogenetic utility of the features of fragmented mt genomes, such as genus-specific minichromosomes and derived gene-arrangement characters.

**Table 4:** **Potential genus-level synapomorphic mitochondrial minichromosomes for the blood-sucking lice investigated to date**

Species	*E-cob-S*_*1*_ *-S*_*2*_	*C-nad6-W-L*_*2*_	*rrnS*	*E-cob-I*	*K-nad4-atp8-atp6-N*	*nad2-I-cox1-L*_*2*_	*D-Y-cox2-S*_*1*_ *-S*_*2*_ *-P-cox3-A*	*E-cob-V*	***Q-nad1*** *-T-G-nad3-W*	*cob*	*R-nad3*	*G-nad4L-V*	*F-nad6*	*L*_*1*_ */L*_*2*_ *-rrnS-C*	*L*_*1*_ *-rrnL*	*S*_*1*_ *-N-E*	*T-D-H*	*W-* *S*_*2*_	*cob* *-S*_*1*_	*G-nad3-V-W-S*_*2*_	*T-D-H-R-nad4L*	*F-nad6-E-M*	*L*_*1*_ *-rrnS*	*C*
*Hoplopleura akanezumi* (rat louse)	+	+	+	–	–	–	–	–	–	–	–	–	–	–	–	–	–	–	–	–	–	–	–	–
*Hoplopleura kitti* (rat louse)	+	+	+	–	–	–	–	–	–	–	–	–	–	–	–	–	–	–	–	–	–	–	–	–
*Polyplax asiatica* (rat louse)	–	–	–	+	–	–	–	–	–	–	–	–	–	–	–	–	–	–	–	–	–	–	–	–
*Polyplax spinulosa* (rat louse)	–	–	–	+	–	–	–	–	–	–	–	–	–	–	–	–	–	–	–	–	–	–	–	–
*Haematopinus suis* (domestic pig louse)	–	–	–	–	+	+	+	+	+	–	–	–	–	–	–	–	–	–	–	–	–	–	–	–
*Haemapinus apri* (wild pig louse)	–	–	–	–	+	+	+	+	+	–	–	–	–	–	–	–	–	–	–	–	–	–	–	–
*Haemapinus asini* (horse louse)	–	–	–	–	+	+	+	+	+	–	–	–	–	–	–	–	–	–	–	–	–	–	–	–
*Pediculus capitis* (human head louse)	–	–	–	–	–	–	–	–	–	+	+	+	+	+	+	+	+	+	–	–	–	–	–	–
*Pediculus humanus* (human body louse)	–	–	–	–	–	–	–	–	–	+	+	+	+	+	+	+	+	+	–	–	–	–	–	–
*Pthirus pubis* (human pubic louse)	–	–	–	–	–	–	–	–	–	–	–	–	–	–	–	–	–	–	+	+	+	+	+	+

Note: neighbor genes are linked by a hyphen “-”; genes in bold, i.e. ***Q-nad1***, have an orientation of transcription opposite to that of other genes; anticodons are: *S*_*1*_: tct, *S*_*2*_: tga, *L*_*1*_: tag, and *L*_*2*_: taa; “+” is for “presence”; “-” is for “absence”.

### Limitations of PCR-based strategies in identifying the complete set of mt chromosomes

The PCR-based strategy we used has its limitations in identifying the complete set of mt chromosomes of the *Hoplopleura* lice. We did not find nine mt genes that were typical of bilateral animals in *Ho. akanezumi*: *trnF*, *trnG*, *trnH*, *trnL*_*1*_, *trnM*, *trnQ*, *nad1*, *nad3* and *nad5*. We did not find *trnF, trnH* and *nad5* either in *Ho. kitti*. These unidentified genes in the *Hoplopleura* lice are present in the fragmented mt genomes of three human lice, two pig lice, the horse louse, and two *Polyplax* rat lice [11–15]. The primer pairs, 249F–249R and 344F–344R (Additional file 1), which we used to amplify the coding regions of the mt minichromosomes of *Ho. akanezumi* and *Ho. kitti* may not be conserved in the chromosomes that contain these unidentified genes. We cannot exclude either the possibility that macrochromosomes or the typical mt chromosome of animals may exist in the *Hoplopleura* rodent lice.

A non-PCR-based, shot-gun strategy was used to sequence the mt genome of the human body louse, *Pediculus humanus*, in conjunction with PCR-based strategy [11, 26]. The shot-gun strategy revealed the mt minichromosomes of the human body louse [11] whereas the PCR-based strategy revealed the less abundant, chimeric mt chromosomes [26]. There is no evidence that the typical mt chromosome of animals is present in the human body louse or other blood-sucking lice. The shot-gun sequencing strategy was possible for the human body louse because of a laboratory strain of this louse that supplied a large number of samples for the sequencing project [11, 27]. For other species of blood-sucking lice, there are no laboratory strains available and it is difficult to collect large number of samples. Thus, PCR-based sequencing strategies become the first option of choice, or the only option for the species of blood-sucking lice collected from wild mammals.

## Conclusions

We sequenced the mt genomes of *Ho. akanezumi* and *Ho. kitti*, collected from the Chevrier’s field mouse, *Apodemus chevrieri*, and the Bower’s white-toothed rat, *Berylmys bowersi*. Both *Ho. akanezumi* and *Ho. kitti* have fragmented mt genomes, with the mt genes that we identified distributed on 10 and 11 minichromosomes, respectively. *Ho. akanezumi* also has a chimeric minichromosome, which has parts of two rRNA genes and a full-length *trnY*_*2*_ gene. These two *Hoplopleura* rodent lice share the same pattern for the distribution of the protein-coding and rRNA genes but differ in tRNA gene content and gene arrangement in four minichromosomes. Like other genera of blood-sucking lice that have been investigated in previous studies, the *Hoplopleura* species have four minichromosomes that are only found in this genus. We conclude that fragmented mt genomes were already present in the MRCA of the two major clades of the blood-sucking lice, which lived ~75 Mya. Intra-genus variation in the pattern of mt genome fragmentation is common in the blood-sucking lice. Such variation provides a novel source of information for inferring genus- and family-level phylogenies of the blood-sucking lice, and for testing the phylogenies inferred from morphology and gene sequences. Future studies should expand into other genera and families of the blood-sucking lice to fully explore the phylogenetic utility of the features of fragmented mt genomes.

### Availability of supporting data

The nucleotide sequences of the mt genomes of the two *Hoplopleura* rodent lice supporting the results of this article have been deposited in GenBank (accession numbers KJ648922–KJ648943).

## Electronic supplementary material

Additional file 1: **PCR primers used to amplify and sequence the mitochondrial genomes of the rodent lice,** ***Hoplopleura akanezumi*** **(** ***Hoa*** **) and** ***Hoplopleura kitti*** **(** ***Hok*** **).** (PDF 92 KB)

Additional file 2: **PCR primers used to verify the mitochondrial minichromosomes of the rodent lice,** ***Hoplopleura akanezumi*** **(** ***Hoa*** **)** **and** ***Hoplopleura kitti*** **(** ***Hok*** **).** (PDF 71 KB)

Additional file 3: **Inferred secondary structures of the mitochondrial tRNAs of** ***Hoplopleura akanezumi*** **(Ha) and** ***Hoplopleura kitti*** **(Hk).** (PDF 136 KB)

Additional file 4: **Alignment of nucleotide sequences of parts of the non-coding regions upstream (A) and downstream (B) of the coding regions of the 10 mitochondrial minichromosomes and a chimeric mitochondrial minichromosomes of** ***Hoplopleura akanezumi.*** 249F and 249R are the PCR primers used to amplify the coding regions of all mitochondrial minichromosomes of *Hoplopleura akanezumi*. (PDF 58 KB)

Additional file 5: **Alignment of nucleotide sequences of parts of the non-coding regions upstream (A) and downstream (B) of the coding regions of the 11 mitochondrial minichromosomes of** ***Hoplopleura kitti.*** 344F and 344R are the PCR primers used to amplify the coding regions of all mitochondrial minichromosomes of *Hoplopleura kitti*. (PDF 255 KB)

## Abbreviations

μl: Microliter
*atp6* and *atp8*: Genes for ATP synthase subunits 6 and 8
bp: Base pair
*cob*: Gene for cytochrome b
DNA: Deoxyribonucleic acid
kb: Kilo base pair
min: Minute
MRCA: Most recent common ancestor
mt: Mitochondrial
Mya: Million years ago
PCR: Polymerase chain reaction
RNA: Ribonucleic acid
rRNA: Ribosomal RNA
*rrnS* and *rrnL*: Genes for small and large subunits of ribosomal RNA
sec: Second
T: Thymine
tRNA: Transfer RNA
tRNA: Transfer RNA
*trnA* or *A*: tRNA gene for alanine
*trnC* or *C*: tRNA gene for cysteine
*trnD* or *D*: tRNA gene for aspartic acid
*trnE* or *E*: tRNA gene for glutamic acid
*trnF* or *F*: tRNA gene for phenylalanine
*trnG* or *G*: tRNA gene for glycine
*trnH* or *H*: tRNA gene for histidine
*trnI* or *I*: tRNA gene for isoleucine
*trnK* or *K*: tRNA gene for lysine
*trnL*_*1*_ or *L*_*1*_: tRNA gene for leucine (anticodon NAG)
*trnL*_*2*_ or *L*_*2*_: tRNA gene for leucine (anticodon YAA)
*trnM* or *M*: tRNA gene for methionine
*trnN* or *N*: tRNA gene for asparagine
*trnP* or *P*: tRNA gene for proline
*trnQ* or *Q*: tRNA gene for glutamine
*trnR* or *R*: tRNA gene for arginine
*trnS*_*1*_ or *S*_*1*_: tRNA gene for serine (anticodon NCU)
*trnS*_*2*_ or *S*_*2*_: tRNA gene for serine (anticodon NGA)
*trnT* or *T*: tRNA gene for threonine
*trnV* or *V*: tRNA gene for valine
*trnW* or *W*: tRNA gene for tryptophan
*trnY* or *Y*: tRNA gene for tyrosine
U: Uracil.
