# Supplementary material for: Fragmented mitochondrial genomes are present in both major clades of the blood-sucking lice (suborder Anoplura): evidence from two Hoplopleura rodent lice (family Hoplopleuridae)
Source: BMC Genomics. 2014 Sep 2;15(1):751. doi: 10.1186/1471-2164-15-751 (PMC4158074; doi:10.1186/1471-2164-15-751)
Supplement: Supplementary file 1 — Additional file 1: PCR primers used to amplify and sequence the mitochondrial genomes of the rodent lice, Hoplopleura akanezumi ( Hoa ) and Hoplopleura kitti ( Hok ). (PDF 92 KB) [file 12864_2014_6419_MOESM1_ESM.pdf]

Additional file 1 – PCR primers used to amplify and sequence the mitochondrial genomes of the rat lice, *Hoplopleura akanezumi* (*Hoa*) and *Hoplopleura kitti* (*Hok*).

**Primer**	**Target Gene**	**Sequence (5' to 3')**	**Species**
12SA	*rrnS*	TACTATGTTACGACTTAT	*Hoa*
12SB	*rrnS*	AAACTAGGATTAGATACCC	*Hoa*
16SF	*rrnL*	TTAATTCAACATCGAGGTCGCAA	*Hoa*
Lx16SR	*rrnL*	GACTGTGCTAAGGTAGCATAAT	*Hoa*
12S 249F	*12S249*	CTATCACCTTCCGACAGCGGTGTACAAGA	*Hoa*
12S249R	*12S249*	AAGGGGATAAGTCAAGTCAAGGTGCAGCC	*Hoa*
16S249F	*16S249*	CAAGATCTATAGGGTCTTCTCGTCCCTCTG	*Hoa*
16S249R	*16S249*	GGAAAGTAATTTTACCTGGGAAGGGGCTC	*Hoa*
249F	*M249*	GGCCTAGGATTAGGGTGGTAGTAATG	*Hoa*
249R	*M249*	CAACCGAGGTTGGGGACAGAAACAAT	*Hoa*
12SA	*rrnS*	TACTATGTTACGACTTAT	*Hok*
12SB	*rrnS*	AAACTAGGATTAGATACCC	*Hok*
16SF	*rrnL*	TTAATTCAACATCGAGGTCGCAA	*Hok*
Lx16SR	*rrnL*	GACTGTGCTAAGGTAGCATAAT	*Hok*
Mtd6	*cox1*	GGAGGATTTGGAAATTGATTAGTTCC	*Hok*
Mtd11	*cox1*	ACTGTAAATATATGATGAGCTCA	*Hok*
12S 344F	*12S344*	CCGACAGCGGTGTACAAGATTTAAGCAAAGG	*Hok*
12S 344R	*12S344*	GGTGATATCCCGTTAACTCACCGTATCCCA	*Hok*
16S 344F	*16S344*	GGTCTTCTCGTCCCTCTGAGACATTTAAGC	*Hok*
16S 344R	*16S344*	GTAGGGATGAATTTTACCTGGGAAGGGG	*Hok*
344cox1F	*344cox1*	GAGAGCATGAGTTAAGATAGA ACTGACTCC	*Hok*
344cox1R	*344cox1*	CACCAGCTATATGGAGCCTGA AAATTGCC	*Hok*
344F	*M344*	GTTGCACTCTATCTGCATAGGATAGGTGCG	*Hok*
344R	*M344*	CCACCCAAAAAGGGTGAGGGGTAGGGTA	*Hok*
