# Supplementary material for: Fragmented mitochondrial genomes are present in both major clades of the blood-sucking lice (suborder Anoplura): evidence from two Hoplopleura rodent lice (family Hoplopleuridae)
Source: BMC Genomics. 2014 Sep 2;15(1):751. doi: 10.1186/1471-2164-15-751 (PMC4158074; doi:10.1186/1471-2164-15-751)
Supplement: Supplementary file 2 — Additional file 2: PCR primers used to verify the mitochondrial minichromosomes of the rodent lice, Hoplopleura akanezumi ( Hoa ) and Hoplopleura kitti ( Hok ). (PDF 71 KB) [file 12864_2014_6419_MOESM2_ESM.pdf]

Additional file 2 – PCR primers used to verify the mitochondrial minichromosomes of the rat lice, *Hoplopleura akanezumi* (*Hoa*) and *Hoplopleura kitti* (*Hok*).

Primer	Target gene	Sequence (5′ to 3′)
249M1F	*trnN*	GCATTGTTAGTGCAATCATAGGATTCTACC
249M1R	*trnN*	CCTCAGCCTATTGGTTTTAACTAATGC
249M2F	*trnS$_1$*	CTAAAGTGATTTGTGCCACTTATACCCC
249M2R	*trnS$_1$*	TTAGAAGTCATGAATAGGTTTCTTACCCCG
249M3F	*cox1*	CAGAGGGAGCTTATTCTTGAACCCCAC
249M3R	*cox1*	GGGAGAGCTCTAATCGAATTAAAATCC
249M4F	*cox2*	GTGTCTAGTGCAGACGTTCTCCACAGATGG
249M4R	*cox2*	GGAACCGAACTTCTGTAAGTAAGGGGATGC
249M5F	*cox3*	CTATTCCTGGGATTCCAGGTAGTTGAGTAC
249M5R	*cox3*	GCAGCCAAGAAGGCATGTGACAAGTAGC
249M6F	*nad2*	GTTATAGCCTTCGTGGAGCTGTTTCCTTC
249M6R	*nad2*	GCAAATTGCGAGAACTCTAAATGTTCGTAG
249M7F	*nad4*	CTCTGAGGCGGCTTGGCTTGACTTTCTTTC
249M7R	*nad4*	CCTCCACTAGAGAGTGGTAAGAATTGATGG
249M8F	*nad6*	GGACTTTTTGTAGTCCCTAGCTCTAACCTC
249M8R	*nad6*	GAGAGAATAACACCTTGGAAGGGCTACAC
249M9F	*rrnS*	AAGGGGATAAGTCAAGTCAAGGTGCAGCC
249M9R	*rrnS*	CTATCACCTTCCGACAGCGGTGTACAAGA
249M10F	*rrnL*	GGAAAGTAATTTACCTGGGAAGGGGCTC
249M10R	*rrnL*	CAAGATCTATAGGGTCTTCTCGTCCCTCTG
249chimericF	*prrnS*	CGCCCGTCACTCCGTATAATAGCGGATAAG
249chimericR	*prrnL*	CAACACAAACGTTGAAACTGTTTACCCTC
344M1F	*trnN*	GGACAGACTCCTAACTACGTTTCTAGGAGG
344M1R	*trnN*	GACCACAGTAACAATGTGGAACCTCAAT
344M2F	*trnE*	GCCTGGAAGAATTGTTGTCTGTAAAG
344M2R	*trnE*	GGACCGTTTACCCAATGAAATTGGAATG
344M3F	*trnI*	GGCGGTTACGGTAGGACATACCTCATAAGG
344M3R	*trnI*	CATCAAAGCGTTCCGTAAACTCCGGCACC
344M4F	*cox2*	GAGGGTTGATAATGAAGTGTGCCTCCC
344M4R	*cox2*	GGGAGCTAGGACTAGTGTTTGATGAGTC
344M5F	*cox3*	CGAGGGGAGTTTCTATTACATGATCCCATC
344M5R	*cox3*	CTCCGATTGGAGGTGTTATACAACCGACCG
344M6F	*nad1*	GGCAACTCCAATTAATATTGCTACTATGGC
344M6R	*nad1*	CCCTCTTTGAACGGAAACTAATAGGAACAG
344M7F	*nad2*	GTGTACTCTTCCATTTTAAGGAGAGGGTG
344M7R	*nad2*	GATATGACGGAGCCTAAAGGATACCAAGG
344M8F	*trnK*	GCTTCGAGATTTTAACTCGACTAAAACC
344M8R	*trnK*	CATAAGCTACCAAGAAATGTGTGAGTTC
344M9F	*nad6*	GAT AGG AGA TTA GCT TGT TCC AGT CAA CTC
344M9R	*nad6*	GTA CAC TCA TTT CAA CTT TCG AGG GGC TAC
344M10F	*rrnS*	GGTGATATCCCGTTAACTCACCGTATCCCA

**Continue-Additional file 2**		
344M10R	*rrnS*	CCGACAGCGGTGTACAAGATTTAAGCAAAGG
344M11F	*rrnL*	GTAGGGATGAATTTTACCTGGGAAGGGG
344M11R	*rrnL*	GGTCTTCTCGTCCCTCTGAGACATTTAAGC

Note: Primers with “249” are for *H. akanezumi*; those with “344” are for *H. kitti*
