# Supplementary material for: Fragmented mitochondrial genomes are present in both major clades of the blood-sucking lice (suborder Anoplura): evidence from two Hoplopleura rodent lice (family Hoplopleuridae)
Source: BMC Genomics. 2014 Sep 2;15(1):751. doi: 10.1186/1471-2164-15-751 (PMC4158074; doi:10.1186/1471-2164-15-751)
Supplement: Supplementary file 3 — Additional file 3: Inferred secondary structures of the mitochondrial tRNAs of Hoplopleura akanezumi (Ha) and Hoplopleura kitti (Hk). (PDF 136 KB) [file 12864_2014_6419_MOESM3_ESM.pdf]

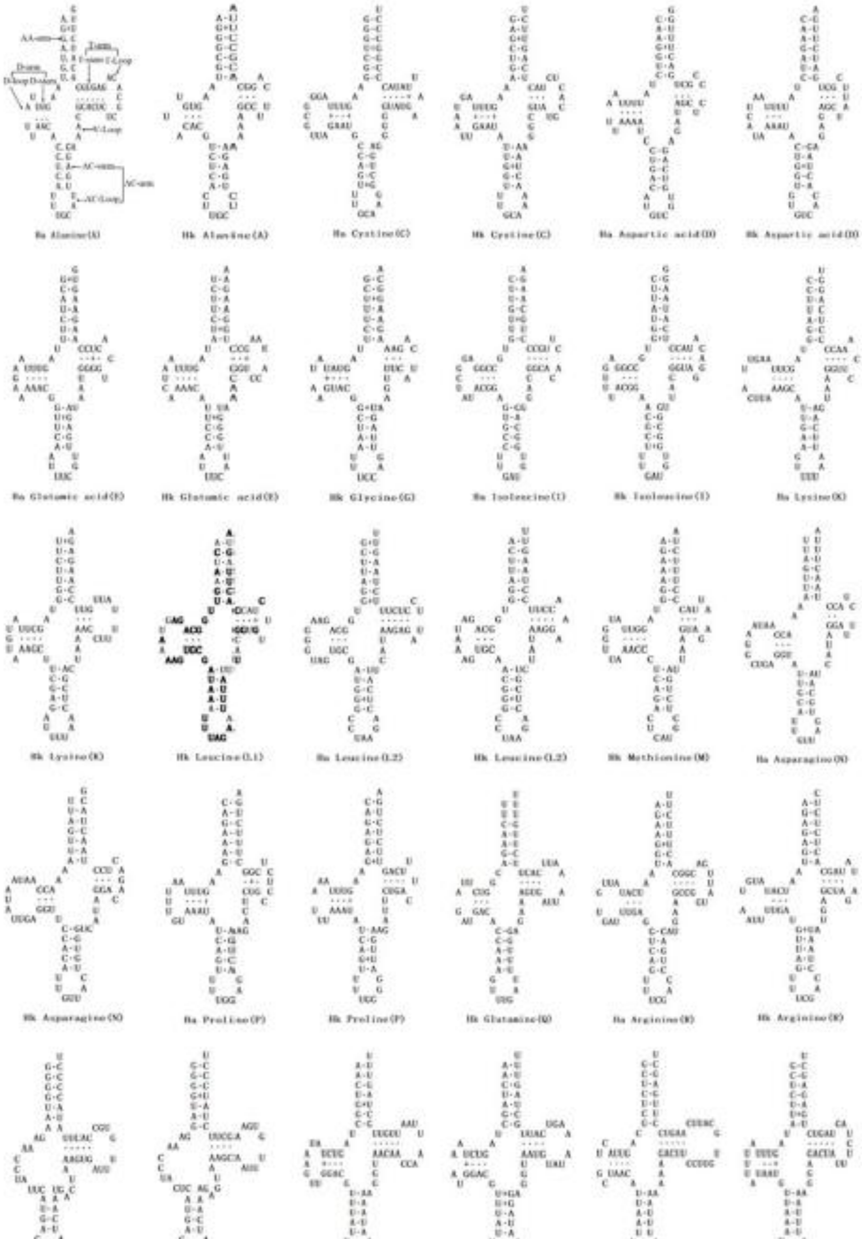

Ha Alanine(A)
Hk Alanine(A)
Ha Cystine(C)
Hk Cystine(C)
Ha Aspartic acid(D)
Hk Aspartic acid(D)
Ha Glutamic acid(E)
Hk Glutamic acid(E)
Hk Glycine(G)
Ha Isoleucine(I)
Hk Isoleucine(I)
Ha Lysine(K)
Hk Lysine(K)
Hk Leucine(L1)
Ha Leucine(L2)
Hk Leucine(L2)
Hk Methionine(M)
Ha Asparagine(N)
Hk Asparagine(N)
Ha Proline(P)
Hk Proline(P)
Hk Glutamine(Q)
Ha Arginine(R)
Hk Arginine(R)
Ha Serine(S1)
Hk Serine(S1)
Ha Serine(S2)
Hk Serine (S2)
Ha Threonine(T)
Hk Threonine(T)

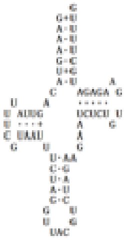

Ha Valine(V)

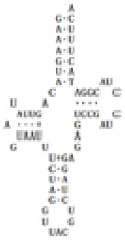

Hk Valine(V)

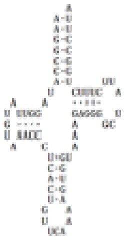

Ha Tryptophan(W)

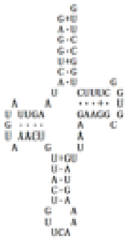

Hk Tryptophan(W)

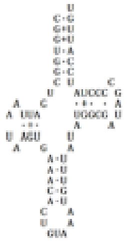

Ha Tyrosine(Y1)

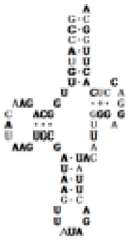

Ha Tyrosine(Y2)

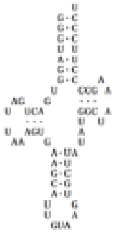

Hk Tyrosine(Y)

Continue-Additional file 5
