# Supplementary material for: Fragmented mitochondrial genomes are present in both major clades of the blood-sucking lice (suborder Anoplura): evidence from two Hoplopleura rodent lice (family Hoplopleuridae)
Source: BMC Genomics. 2014 Sep 2;15(1):751. doi: 10.1186/1471-2164-15-751 (PMC4158074; doi:10.1186/1471-2164-15-751)

Additional file 3 (A)

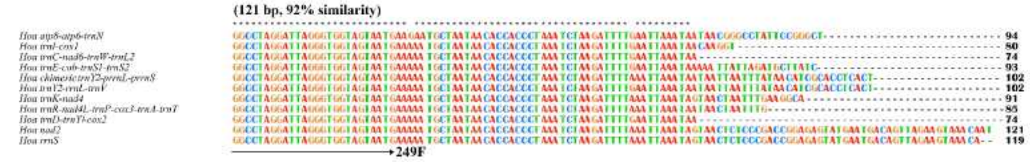

Additional file 3 (B)

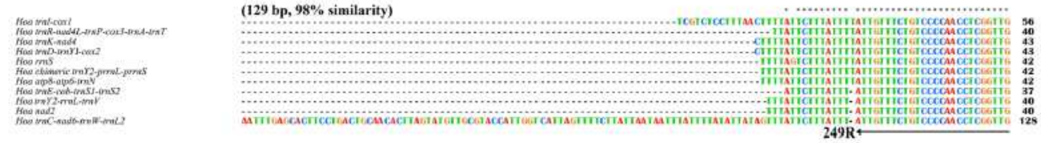

Supplement: Supplementary file 4 — Additional file 4: Alignment of nucleotide sequences of parts of the non-coding regions upstream (A) and downstream (B) of the coding regions of the 10 mitochondrial minichromosomes and a chimeric mitochondrial minichromosomes of Hoplopleura akanezumi. 249F and 249R are the PCR primers used to amplify the coding regions of all mitochondrial minichromosomes of Hoplopleura akanezumi. (PDF 58 KB) [file 12864_2014_6419_MOESM4_ESM.pdf]
