# Supplementary material for: Fragmented mitochondrial genomes are present in both major clades of the blood-sucking lice (suborder Anoplura): evidence from two Hoplopleura rodent lice (family Hoplopleuridae)
Source: BMC Genomics. 2014 Sep 2;15(1):751. doi: 10.1186/1471-2164-15-751 (PMC4158074; doi:10.1186/1471-2164-15-751)

Additional file 4 (A)

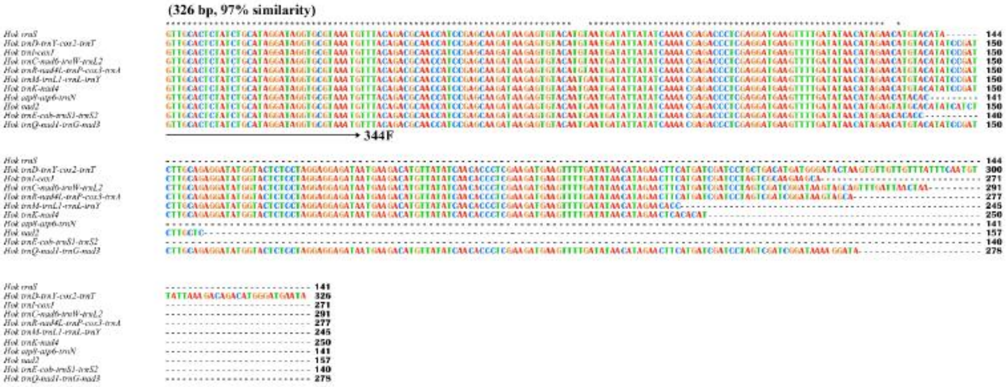

Additional file 4 (B)

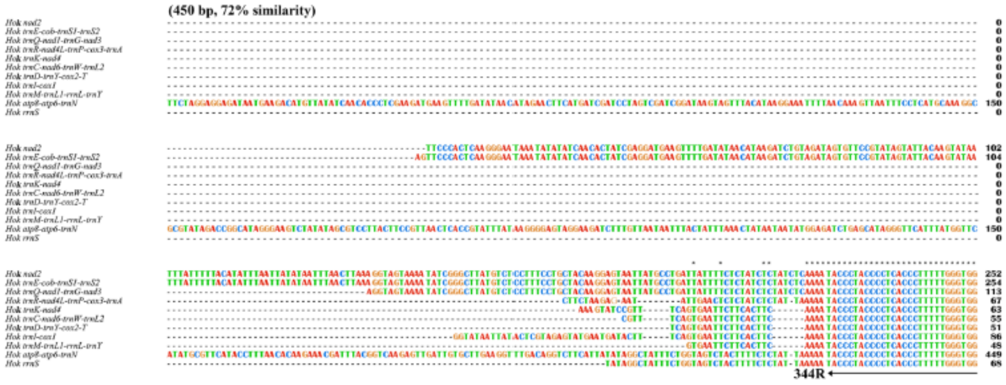

Supplement: Supplementary file 5 — Additional file 5: Alignment of nucleotide sequences of parts of the non-coding regions upstream (A) and downstream (B) of the coding regions of the 11 mitochondrial minichromosomes of Hoplopleura kitti. 344F and 344R are the PCR primers used to amplify the coding regions of all mitochondrial minichromosomes of Hoplopleura kitti. (PDF 255 KB) [file 12864_2014_6419_MOESM5_ESM.pdf]
